# A case of focal segmental glomerulosclerosis in an adult patient with hypogammaglobulinemia superimposed on membranoproliferative glomerulonephritis in childhood

**DOI:** 10.1186/1471-2369-13-46

**Published:** 2012-06-24

**Authors:** Kenji Tsuji, Haruhito Adam Uchida, Tetsuichirou Ono, Tatsuyuki Inoue, Katsuji Shinagawa, Shinji Kitamura, Yohei Maeshima, Hitoshi Sugiyama, Hirofumi Makino

**Affiliations:** 1 Department of Medicine and Clinical Science, Okayama University Graduate School of Medicine, Dentistry and Pharmaceutical Sciences, 2-5-1 Shikata-cho, Okayama, kita-ku 700-8558, Japan; 2 Department of Hematology and Oncology, Okayama University Graduate School of Medicine, Dentistry and Pharmaceutical Sciences, 2-5-1 Shikata-cho, Okayama, kita-ku, 700-8558, Japan

**Keywords:** Hypogammaglobulinemia, Focal segmental glomerulosclerosis, Proteinuria

## Abstract

**Background:**

Common variable immunodeficiency (CVID) is a disorder characterized by hypogammaglobulinemia without a known predisposing cause.

**Case presentation:**

We report a 36-year-old man who had suffered membranoproliferative glomerulonephritis (MPGN) in his childhood, later diagnosed with CVID at 35 years of age. He presented at our hospital with signs of proteinuria. A renal biopsy revealed he suffered from focal segmental glomerulosclerosis (FSGS), possibly due to obesity and hypertension, not CVID - associated MPGN.

**Conclusion:**

This is the first case report of FSGS in a CVID patient. In this case, we have to pay attention not only to the treatment of obesity and hypertension for FSGS but also to the recurrence of immune-complex glomerulonephritis such as MPGN, in case of the restoration of hypogammaglobulinemia.

## Background

Patients with hypogammaglobulinemia seem to be less susceptible to immune complex- and complement- related nephritis, such as MPGN, membranous glomerulonephritis (MN), and lupus nephritis. Indeed, there have been few reports of glomerular nephritis in patients with hypogammaglobulinemia, with the exception of three cases whom developed glomerular nephritis during intravenous immunogloburin (IVIG) treatment [1-3].

FSGS is one of the glomerulonephritis which is characterized by podocyte damage. The pathologic classification of FSGS is categorized as primary and secondary, the latter being caused by human immunodeficiency virus (HIV)-associated, heroin, familial forms, drug toxicities, as well as structural-functional adaptations to glomerular hyperfiltration such as obesity, hypertension, reflux nephropathy, etc. Detailed mechanisms for the development of this disease are still unclear, but Changli Wei et al. found recently that a circulating, soluble form of the urokinase receptor (suPAR) can activate podocyte b3 integrin, leading to FSGS pathology [4].

Here, we report a case of hypertension and obesity-related FSGS in a CVID patient who had suffered MPGN in his childhood.

## Case presentation

The patient in this report was a 36-year-old Japanese man. He had a history of continuation of nephrotic proteinuria and microscopic hematuria since the age of 7 years. Although information on the therapy and progress of these diseases were not fully available, glucocorticoid and other immunosuppressant therapy were initiated at the age of 7 years in a hospital. Renal biopsy was performed at the age of 14 years, with the diagnosis of MPGN. Immunosuppressant therapy was terminated due to the absence of active findings in the glomeruli. Microscopic hematuria gradually disappeared whereas mild proteinuria continued.

Because of recurrent bacterial infections at 35 years of age, he consulted his doctor, and initial testing revealed low levels of serum IgG, IgA, and IgM. After a detailed examination by our on-site hematologist, including bone marrow examination and genetic analysis, he was diagnosed with CVID although he had no family history of recurrent infections or immunoglobulin deficiencies. Since his serum IgG level was not low enough to be considered unsafe, there was no follow-up therapeutic treatment including IVIG. He was also diagnosed with hypertension and advised to start the treatment with an antihypertensive drug, but he refused at that time. It should also be noted that during the detailed examination for the diagnosis of CVID, papillary thyroid carcinoma happened to be found; surgery was recommended immediately and a thyroidectomy was performed.

At the age of 36 years, his primary physician diagnosed increased proteinuria [over 1.0 g/day (U-pro/Cr : 1.3 g/gCr)], and the patient thereafter came to our hospital. At the first visit, he presented with mild proteinuria (U-pro/Cr : 0.61 g/gCr) and glomerular hyperfiltration (24hCCr : 175.4 ml/min). Two months later, he was admitted to our hospital the renal biopsy because of mild, persistent proteinuria.

**Figure 1:**
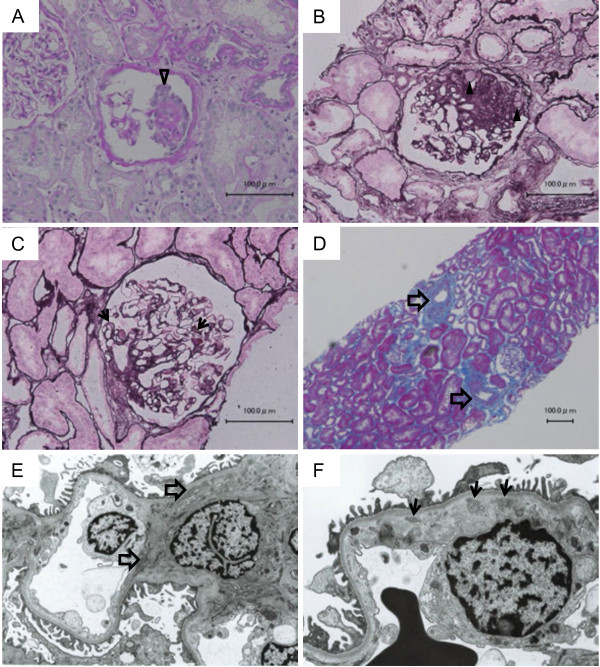
**Light microscopic analysis (A-D) and electron microscopy analysis (E,F) Light microscopic analysis (A-D) and electron microscopy analysis (E,F).A** : A glomerulus showed podocyte hyperplasia (open arrowhead). Periodic acid-Schiff stain. **B** : Segmental glomerulosclerosis in perihilar lesion was observed in two glomeruli. This glomerulus contained perihilar hyalinosis (arrowheads). Periodic acid silver methenamine stain. **C** : Many glomerulus showed double contours of capillary walls (arrows). Periodic acid silver methenamine stain. **D** : Mild arteriolosclerosis were found (open arrows). Masson trichrome stain. **E** : Electron-dense deposits was observed within the mesangial and para-mesangial region (open arrows). **F** : A few electron-dense deposits were found within subendothelial space (arrows), but their densities looked sparser than typical dense deposits, implying that these deposits appeared to be formed previously.

On admission, the patient’s height was 171.8 cm, body weight was 81.6 kg, body mass index was 28.2 kg/m^2^, blood pressure was 115/85 mmHg, body temperature was 36.4°C. His cardiac and pulmonary function appeared normal and no edema was seen. He snored terribly, implying that he had obstructive sleep apnea. Laboratory data upon admission is shown in Table1. Serologic testing for HIV-1/HIV-2 was negative. Renal biopsy was performed at day 2. Immunofluorescence microscopy showed no specific staining of immunogloburin, complement, or fibrinogen. Histopathology showed that obtained glomeruli were enlarged (max and mean diameter of glomeruli was 270 and 220 μM). Double contours of capillary walls and mild arteriolosclerosis were found. Segmental glomerulosclerosis in perihilar lesion was observed in two glomeruli, one of which contained perihilar hyalinosis and podocyte hyperplasia (Figure1). In electron microscopic observation, electron-dense deposits were visible within the mesangial, para-mesangial, and subendotherial space, but the density of the subendotherial deposits appeared sparser than typical dense deposits, implying that these deposits appeared to be formed previously (Figure1). Given these observation, the patient was diagnosed as FSGS, possibly due to obesity and hypertension. At the same time, renal biopsy showed previous MPGN.

**Table 1:** Laboratory data on admission

**Blood cell count**				**Urinalysis**	
WBC	7800/μL	Na	139 mEq/L	Specific gravity	1.020
RBC	571 × 10^4^/μL	K	4.6 mEq/L	PH	5.5
Hb	16.3 g/dl	Cl	104 mEq/L	Protein	1+
Ht	48.6%	Ca	9.2 mg/dL	Occult blood	-
Plt	37.1 × 10^4^/μL	P	3.2 mg/dL	Glucose	-
		HbA1c	6.0%	RBC	<1/HPF
Blood chemistry		CRP	0.14 mg/dl	WBC	<1/HPF
TP	6.5 g/dL			Cast	-
Alb	4.5 g/dL	Serological test		NAG	23.4 U/mL
AST	17 IU/L	IgG	473.4 mg/dL	β_**2**_M	0.100 mg/L
ALT	25 IU/L	IgA	118.5 mg/dL		
T-cho	194 mg/dL	IgM	35.5 mg/dL	U-protein	0.45 g/day
LDL-cho	128 mg/dL	C3	114.7 mg/dL	24hCCr	131.9 ml/min
HDL-cho	33 mg/dL	C4	26.9 mg/dL		
UA	6.9 mg/dL	CH50	49 U/mL		
BUN	11.3 mg/dL	ANA	×7.8		
Cr	0.86 mg/dL				

*WBC* : white blood cell, *RBC* : red blood cell, *Hb* : hemoglobin, *Ht* : hematocrit, *Plt* : platelet, *TP* : total protein, *Alb* : albumin, *AST* : aspartate aminotransferase, *ALT* : alanine aminotransferase, *T-cho* : total cholesterol, *LDL-cho* : low-density lipoprotein cholesterol, *HDL-cho* : high-density lipoprotein cholesterol, *UA* : uric acid, *BUN* : blood urea nitrogen, *Cr* : creatinine, *Na* : sodium, *K* : potassium, *Cl* : chloride, *Ca* : calcium, *P* : phosphorus, *HbA1c* : hemoglobin A1c, *CRP* : cross-reactive protein, *IgG* : immunoglobulin G, *IgA* : immunoglobulin A, *IgM* : immunoglobulin M, *C3* : complement C3, *C4* : complement C4, *CH50* : hemolytic complement, *ANA* : antinuclear antibody, *NAG* : N-acetyl-β-glucosaminidase, *β*_***2***_*M* : β_**2**_microglobulin, *U-protein* : urinary-protein, *24hCCr* : 24-hour endogenous creatinine clearance.

## Conclusions

CVID is a disorder characterized by hypogammaglobulinemia without a known, predisposing cause [5]. Meanwhile, immune complex and complement systems are thought to play an important role on the pathogenesis of nephritis, such as MPGN and MN. Since immunodeficiency diseases, such as CVID and X-linked agammaglobulinemia, cause extremely low levels of immunoglobulin, patients diagnosed with these seem to have low incidence of MPGN or MN. Indeed, as far as we know, there have been only three previous case reports of nephritis associated with hypogammaglobulinemia due to immunodeficiency diseases. Two of these cases were MPGN and one was MN. In each case, nephritis was developed during IVIG treatment [1-3]. On the other hand, there is a paradoxical relationship between immunodeficiency diseases and autoimmune diseases. For example, Barnett et al. has reported that X-linked agammaglobulinemia is associated with polyarticular arthritis and inflammatory bowel diseases. Barnett et al. has also reported that CVID is associated with rheumatoid arthritis, pernicious anemia, and hemolytic anemia [6-8]. The precise mechanism linking CVID with these diseases still remains unclear.

FSGS, clinical-pathologic syndrome of proteinuria associated with focal and segmental sclerotic glomerular lesions, is characterized by podocyte damage. The pathologic classification of FSGS is categorized as primary and secondary, the latter of which is caused by HIV-associated, heroin, familial forms, drug toxicities, but also by structural-functional adaptations to glomerular hyperfiltration such as obesity, hypertension, reflux nephropathy, and so on. A number of morphologic variants of primary and secondary focal sclerosis are now recognized, based on a 2004 Columbia classification system, including FSGS-not otherwise specified (NOS), perihilar, cellular, tip, and collapsing variants. Recently, Changli Wei et al. found that a circulating, soluble form of the urokinase receptor (suPAR) can activate podocyte b3 integrin, leading to FSGS pathology, which provides new insight into this disease and may have important clinical implications [4].

In our case, differential diagnosis before renal biopsy appeared somewhat difficult because this patient had suffered MPGN in his childhood. In addition, the patient had many complications such as obesity, hypertension, post operative-papillary thyroid carcinoma, and CVID. We considered several possibilities, recurrence of MPGN, CVID-related nephritis, malignancy related nephritis, etc. Renal biopsy finally revealed this patient to have FSGS. According to The Columbia FSGS Classification, this patient was categorized as perihilar variant which was normally due to glomerular hypertension [9]. Namely, it is likely that metabolic disorder, relative to his obesity and hypertension, might have “encouraged” the development of FSGS. Renal biopsy also implied previous MPGN at the same time. There was no clear relationship between our findings and the patient’s chronic, present proteinuria. Since his episode of past MPGN was successfully treated, we could find no link between MGPN and CVID for this case. We started his treatment with diet, exercise, angiotensin receptor blocker (ARB), and statin therapy.

Since hypogammaglobulinemia due to CVID may disturb immune complex and complement system, there is a possibility that current CVID prevents the development of MPGN. In case of recovery of CVID, restoration from hypogammaglobulinemia may cause the recurrence of MPGN through the activation of immune complex and complement system. Therefore, in the present case, we have to pay attention not only to the treatment of obesity and hypertension for FSGS but also to the recurrence of immune-complex related glomerulonephritis such as MPGN.

In conclusion, we report for the first time a case of CVID patient with FSGS. Detection of proteinuria in a CVID patient is rare. In the case of CVID patient with proteinuria, we have to take many possibilities into consideration. Therefore, renal biopsy should be an important tool for a precise and proper diagnosis.

## Consent

Written informed consent was obtained from the patient for publication of this Case report and any accompanying images. A copy of the written consent is available for review by the Series Editor of this journal.

## Abbreviations

CVID: Common variable immunodeficiency; MPGN: Membranoproliferative glomerulonephritis; FSGS: Focal segmental glomerulosclerosis; MN: Membranous glomerulonephritis; IVIG: Intravenous immunogloburin; HIV: Human immunodeficiency virus; suPAR: Soluble form of the urokinase receptor; NOS: Not otherwise specified; ARB: Angiotensin receptor blocker.

## Competing interests

The authors declare that they have no competing interests.

## Authors’ contributions

KT, HAU, TO and KS were the treating physicians of the patient reported. KT, HAU, TI, SK, YM, HS and HM performed the evaluation of the renal biopsy. All authors participated in the discussion of the manuscript and approved the final version.

## Pre-publication history

The pre-publication history for this paper can be accessed here:

http://www.biomedcentral.com/1471-2369/13/46/prepub
